# Making evidence-based approaches to autism accessible

**DOI:** 10.2471/BLT.17.030817

**Published:** 2017-08-01

## Abstract

One of the greatest challenges for the public health response to autism is providing access to evidence-based care. Sally J Rogers tells Andréia Azevedo Soares how parents can help their children mitigate the disabilities associated with autism.

Q: What is autism and how does it affect children and their families?

A: Autism spectrum disorders cover a range of symptoms leading to moderate to severe disabilities, including difficulties with social communication and reciprocal exchanges with others, and repetitive behaviours. There are often delays in communication and language, learning and motor skills.

Q: How did you become interested in these disorders?

A: As a teenager I read an article in *Life *magazine about how children with autism do not interact with other people and found this intriguing. My first experience with such children was as an undergraduate psychology student. These children had been living in institutions and had not received proper treatment, so their signals were very subtle and they did not understand how people operated and communicated. In spite of that, they were not unresponsive. I was keen to find out how much they could learn to interact with others, so that they would become less disabled by their disorder. Then, in 1981, while working in Denver at the JFK Center of the University of Colorado Medical School, I received a federal grant to start a pre-school intervention programme for children with autism and their families.

Q: Can you tell us about this programme which became known as the Denver Model, one of several evidence-based approaches to autism?

A: It was a treatment programme for pre-schoolers with autism in which therapists worked with children aged 3 to 5 years in small groups to help them develop communication, language, play and social skills using typical pre-school activities for this age group. We also shared these activities with parents to support their children’s learning at home. For example, parents position themselves face-to-face with the child during floor-time play and try to gain the child’s attention through the child’s interests, such as toy animals. The approach was based on the science of child development and it applied the principles of how young children usually acquire these skills. At the time, in the 1980s, it was assumed that children with autism spectrum disorders were unable to learn through play and other daily activities e.g. mealtime, bathtime, books before bedtime. Our work in Denver helped us understand how to apply developmental and learning science concepts to children with these disorders.

Q: Can you tell us about your work on an approach for younger children?

A: In 2003, I joined Geraldine Dawson, who is now a professor at Duke University, at the University of Washington in her large project to develop an intervention for even younger children, aged 12 to 30 months. This became the Early Start Denver Model (ESDM). The approach integrates developmental and learning sciences to treat the symptoms of these disorders in toddlers, such as delayed speech. The approach is a routines and play-based therapy that takes place in children’s natural environment, i.e. in their homes or care centres – and embeds learning opportunities into a child’s daily routines. It is a flexible intervention that can be delivered in different places (home, pre-school or day-care settings) in various formats (one-on-one, group) and by various people (parents, caregiver, therapists). We also taught parents intervention techniques to incorporate into their home routines so that children were receiving high quality learning opportunities from waking until bedtime.

Q: What challenges did you face?

A: The misconception that children with these disorders learn in fundamentally different ways to other children. Developmental science studies show that on the contrary young children with these disorders follow the same developmental paths as those with other developmental disabilities or no disability, but often at a slower pace than their peers. This means that although they learn and develop differently to other children we don’t need to create special learning settings, interactions and materials for them. 

*Q: In your co-authored 2012 book, *An early start for your child with autism,* you provide parents with the same tools therapists use. Do you see parents as therapists too?*

A: All parents teach their children the critical things in life. A child’s development is set in the first five years. If parents can learn to help children with these disorders to learn in everyday interaction, their children will receive the best treatment possible. When we teach parents these techniques in the USA, they often say: "This is similar to what I do with all my kids". The difference is that the parent or therapist needs to work hard to gain and hold the child’s attention, and to break learning, especially language learning, down into small steps.

“We are tapping into some universal values.”

Q: How universally applicable are these techniques?

A: When I first started teaching these techniques outside of the USA, I worried that our approach might be too American and would not translate to other cultures. But the feedback I have received from our work with parents and therapists in Australia, China, India, the Philippines, South Africa, Thailand, Viet Nam and many countries in Europe, has shown that this approach is considered appropriate and acceptable in many cultures and that children respond well to them. I guess we are tapping into some universal values and practices in the way that parents and children relate to each other.

Q: Specialist training is expensive even in high-income countries. Have you applied your approach in low- and middle-income countries?

A: Yes. I was involved in a project in South Africa several years ago with colleagues from the University of Cape Town and Duke University to do research on how to make these interventions more widely available. We have three approaches. One, we train pre-school specialists to embed our approach into pre-school curricula. Two, we train professionals working in different disciplines to use the techniques themselves in their treatment sessions with children. And three, we teach those who work with families to coach parents to incorporate the treatment into everyday activities with their children. Finally, we are experimenting with coaching families in these techniques remotely via the Internet using mobile devices.

Q: How could smartphones help?

A: When I was in a township in Cape Town with a single mother of a four-year-old boy who was severely affected by these disorders and a four-week-old baby on her hip, I realized there were thousands of parents in such situations without access to specialized therapy. It occurred to me that if our materials were available online, this mother could access them via her smartphone. My colleagues and I have created materials for such parents accessible on their smartphones in lessons lasting less than 10 minutes. The lessons are described in our book, *An early start for your child with autism*, but presented in a more accessible way through cartoons and videos. We are field testing the intervention and hope it will be available in a couple of years.

Q: What effect has the 1998 study falsely linking the measles–mumps–rubella (MMR) vaccine to autism had on autism research? How have you and your colleague re-built confidence in autism spectrum disorders science?

A: This underscores the importance of taking an evidence-based approach, something I and my colleagues have always done. Our ESDM approach, for example, is a scientifically rigorous approach that has been tested in observational studies published in peer-reviewed journals. One way we try to build confidence is to provide information about the harm that untested treatments can do, either through actual adverse effects, or by replacing an evidence-based treatment.

Q: Although the paper was rebutted scientifically and retracted, some people still believe that vaccines have created an autism epidemic. What is the evidence-based explanation for the increase in the incidence and prevalence of these disorders in recent years?

A: More children are being diagnosed now than a few decades ago because of greater awareness, through increased scientific literature and media coverage, and changes in the diagnostic criteria, such as recognizing milder symptoms, which result in more children in the autism spectrum. In addition, some parents and practitioners emphasize an autism spectrum diagnosis more today. For instance, a child with intellectual disability and autism spectrum disorder is more likely to have the autism diagnosis prioritized in the USA; 10−20 years ago more weight would have been placed on the intellectual disability diagnosis.

Q: To what extent can early intervention reduce the burden on individuals and society due to autism spectrum disorders?

A: Many adults with autism will need care and financial support throughout their lives. There is an emotional and financial burden on the family. Many adults with these disorders experience depression, anxiety, isolation and health difficulties. However, David Mandell's work in the USA suggests that intensive, early intervention of high quality is cost-effective, with the net savings occurring soon after intensive early intervention ends. While our approach costs more to deliver than some community interventions during the first two years, it appears to pay for itself in terms of reduced needs for therapy and educational support during later childhood. These models suggest that further savings to society will accrue due to more employment in adulthood.

“These children are less likely to be marginalized and untreated.”

*Q: Recently the United Nations established a World Autism Awareness Day. In 2014, the World Health Assembly called for a better response to autism spectrum disorders, which are included in *WHO’s Comprehensive mental health action plan 2013–2020*. WHO is now working in 20 countries to help address these disorders. Has awareness of autism increased? *

A: There is much greater awareness. This means these children are less likely to be marginalized and untreated and we can start interventions as soon as we see that a child is having developmental delays. All children need to be educated, including those with autism spectrum disorder. The earlier we treat their symptoms, the better they will be prepared to go to school. We can now detect high risk for autism by the first birthday and often diagnose children by the time they are two. This is why the paediatric community is making greater efforts to identify developmental and learning disabilities as early as possible so that children can receive the appropriate care and interventions.

